# Functional conduction system mapping in sheep reveals Purkinje spikes in the free wall of the right ventricular outflow tract

**DOI:** 10.3389/fphys.2025.1631426

**Published:** 2025-07-01

**Authors:** Michiel Blok, Bram L. den Ouden, Marion Kuiper, Daan R. M. G. Ophelders, Monique R. M. Jongbloed, Stef Zeemering, Bjarke Jensen, Arne van Hunnik, Bastiaan J. Boukens

**Affiliations:** ^1^ Department of Anatomy and Embryology, Leiden University Medical Center, Leiden, Netherlands; ^2^ Department of Medical Biology, Amsterdam Cardiovascular Sciences, Amsterdam University Medical Center, location Academic Medical Center, Amsterdam, Netherlands; ^3^ Laboratory of Experimental Cardiology, Department of Cardiology, Leiden University Medical Center, Leiden, Netherlands; ^4^ Department of Microelectronics, Delft University of Technology, Delft, Netherlands; ^5^ Department of Physiology, Cardiovascular Research Institute Maastricht, Maastricht University, Maastricht, Netherlands; ^6^ Department of Pediatrics, Maastricht University Medical Center, MosaKids Children’s Hospital, Maastricht, Netherlands; ^7^ GROW Research Institute for Oncology and Reproduction, Maastricht University, Maastricht, Netherlands; ^8^ Department of Cardiology, Centre for Congenital Heart Disease Amsterdam-Leiden (CAHAL), Leiden University Medical Center, Leiden, Netherlands

**Keywords:** right ventricular outflow tract, cardiac Purkinje system, cardiac arrhythmia, cardiac electrophysiology, cardiac conduction system

## Abstract

Ablation of sites displaying Purkinje activity is highly effective against idiopathic ventricular fibrillation which often originates in the right ventricular outflow tract. However, during endocardial mapping Purkinje potentials are rarely, if never, detected in the right ventricular outflow tract. In the present study, we aimed to determine whether the Purkinje system extends into the right ventricular outflow tract. Hearts of five female sheep were blood-perfused in a Langendorff setup in which we performed epicardial and endocardial voltage mapping. During atrial pacing, the right ventricular outflow tract epicardium activated later than the epicardium of the left and right ventricular free walls. Endocardial mapping revealed Purkinje spikes at several sites in the free wall of the right ventricular outflow tract. In one heart, Purkinje spikes preceded ventricular premature beats during mapping, but were not visible during sinus rhythm. Subsequent immuno-histological examination showed a network of Connexin 40-positive Purkinje fibers across and within the wall of the right ventricular outflow tract. Quantitative analysis revealed that the transmural Purkinje fiber network was more abundant near the endocardium than epicardium. In conclusion, the Purkinje system extends into the right ventricular outflow tract of the sheep heart. These findings demonstrate that the sheep could be a valuable model for studying Purkinje-related arrhythmias in the right ventricular outflow tract.

## Introduction

The cardiac Purkinje system is made up of fast-conducting fibers, which are responsible for an orderly propagation of electrical impulses through the ventricles and ensure synchronous contractions of the left and right ventricles (Boyden et al., 2010; Tawara, 1906). While much of the Purkinje system has been well-characterized in various species, including the sheep heart, whether it extended into the right ventricular outflow tract (RVOT) was often not determined (Tawara, 1906; Lhamon, 1912; King, 1916; Hondeghem and Stroobandt, 1974; Canale et al., 1983; Ansari et al., 1999; Ryu et al., 2009). Given that the RVOT activates later during the cardiac cycle than the left and right ventricles, it is conceivable that it would be devoid of a fast-conducting Purkinje system.

The Purkinje system is frequently the substrate for life-threatening ventricular arrhythmias, including monomorphic and polymorphic ventricular tachycardias, as well as ventricular fibrillation (Haissaguerre et al., 2016). In patients with idiopathic ventricular fibrillation, sites showing Purkinje spikes serve as a target for electrogram-based catheter ablation (Haïssaguerre et al., 2002a; Haïssaguerre et al., 2002b; Coronel et al., 2021). In some cases, the Purkinje system itself can also be the origin of arrhythmia, for example, in patients suffering from ventricular premature beats (VPBs). Similar to the Purkinje system, the RVOT is often the origin of ventricular arrhythmia, including idiopathic ventricular fibrillation and VPBs (Enriquez et al., 2024). Therefore, determining whether the Purkinje system extends into the RVOT would provide valuable insight into the origin of VPBs and potentially the arrhythmia mechanisms underlying idiopathic ventricular fibrillation originating from the RVOT.

Evidence for the presence of a Purkinje system in the human RVOT comes from a single study in which conventional histological stains were used on material from two hearts, revealing a few subendocardial bundles of myocardium somewhat insulated by fibrous tissue (De Almeida et al., 2020). A subsequent study investigated two bovine hearts and found that the Purkinje system extends beyond the subendocardium and deeper into the myocardium of the RVOT (De Almeida et al., 2017; De Almeida et al., 2021). Building upon Jan Purkinje’s initial findings in the sheep heart (Tawara, 2000), our study aimed to establish whether the Purkinje system extends into the RVOT. This was achieved through epicardial and endocardial voltage mapping of the RVOT free wall in Langendorff-perfused sheep hearts, as well as immunohistochemical detection of Connexin 40. Overall, our findings support the presence of a functional Purkinje system in the sheep RVOT, making it a suitable model for studying RVOT-related arrhythmias.

## Methods

### Animal approval

Hearts were explanted from sheep that had just undergone caesarean section to deliver near-term fetuses as part of a separate investigation. This was conducted in accordance with national and institutional guidelines, in compliance with the European Commission Directive, and was approved by the local Animal Experiments Committee (approval number: AVD10700202216526).

### Langendorff experiments

Five female sheep (approximately 40 kg) were premedicated with zolazepam and atropine, and received propofol for induction and a combination of propofol and fentanyl for maintenance of anesthesia. The animal was euthanatized with pentobarbital. Before explanting the heart, 25,000 IU heparin was intravenously administered and approximately 600 mL of blood was collected from the femoral artery. Ventricular fibrillation was induced by touching the right ventricular myocardium with the poles of a 9V battery. During explantation, the heart was rapidly excised and submerged in ice-cold Tyrode’s solution. The aorta was then cannulated and mounted on a Langendorff perfusion setup. The heart was then perfused with around 1.4 L of recirculating blood-Tyrode’s mixture (1:1) that was gassed with 95% O_2_/5% CO_2_ and kept at room temperature by running the perfusate through a coil shaped glass heat exchanger. After an equilibration period, the heart was defibrillated using a single direct current shock of 20 J. A reference electrode was connected to the aortic root. Both the reference electrode and recording multi-electrode were connected to an ActiveTwo acquisition setup (BioSemi, Amsterdam, Netherlands). Recordings were performed at a sampling rate of two or 16 KHz. For simultaneous unipolar electrogram recordings of the RVOT epicardium and endocardium, we used an 8.5 × 6.5 mm large 13 × 16 electrode grid (0.5 mm interelectrode distance) and a 4 × 3.7 mm large 3 × 3 + 1 electrode grid (0.6–0.7 mm interelectrode distance), respectively. Data was analyzed using custom-made software based on Matlab R2021a (Mathworks Inc., Natick, MA). Local activation time was determined using the maximal -dV/dt from the unipolar electrogram. Activation was measured in relation to the onset of the earliest ventricular (Purkinje or working myocardium) deflection within the entire electrode grid.

### Histology and immunohistochemistry

After Langendorff experiments, the RVOT free wall was dissected and fixed 24 h in 4% paraformaldehyde and embedded in paraffin. Tissues were longitudinally sectioned 7 μm-thick and mounted on glass slides. Prior to staining, sections were deparaffinised by incubating twice in xylene followed by rehydration through a series of graded ethanol (EtOH) steps (100% EtOH - 100% EtOH - 90% EtOH - 80% EtOH - 70% EtOH) into demineralized H_2_O.

For immunofluorescence staining, sections were subjected to heat-induced antigen retrieval in citric acid buffer (10 mM citric acid, 0.05% Tween 20, pH 6.0) for 12 min at 97°C using the Shandon TissueWave 2 (Thermo Fisher Scientific). Next, sections were rinsed twice with PBS and once with PBS containing 0.02% Tween 20 (PBS-T) and incubated overnight at room temperature with IgG primary antibody against human Connexin 40 (1 μg/mL; Santa Cruz Biotechnology Inc.; sc-20466) in blocking solution containing PBS-T and 1% bovine serum albumin. After rinsing twice with PBS and once with PBS-T, sections were incubated for 1 h at room temperature with Alexa Fluor 647 donkey anti-goat IgG secondary antibody (10 μg/mL; Invitrogen; A-21447). Afterwards, sections were rinsed twice with PBS and once with PBS-T and incubated for 10 min at room temperature with DAPI (5 μg/mL; Invitrogen; D3571). Finally, sections were rinsed twice with PBS and mounted in ProLong Gold Antifade Mountant (Invitrogen; P36930). Super-resolution imaging was done with the ZEISS LSM 900 with Airyscan.

Picrosirius Red staining, cross-stained with Weigert’s Haematoxylin, was used to visualize collagenous tissue in red, cytoplasm in yellow, and nuclei in black. For quantitative analysis, stainings were performed on 19 sections taken throughout the entire thickness of the RVOT free wall (N = 2). Briefly, rehydrated sections were incubated with Weigert’s Haematoxylin for 10 min followed by 10 min of washing in running tap water. Next, sections were incubated with Picrosirius Red for 1 h. Subsequently, sections were rinsed twice with acidified water and rapidly dehydrated trice by dipping 5 times in EtOH 100%. Finally, sections were cleared by three incubation steps of 2 min each with xylene and mounted in Entellan. Brightfield images were captured with the 3DHistech Pannoramic 250 slidescanner (3DHISTECH, Hungary).

### Purkinje fiber quantification

Individual Purkinje fibers were counted within 0–5 mm, 5–10 mm, and 10–15 mm below the hinge-line of the pulmonary valvular leaflets. The shortest Purkinje fiber distance relative to the endocardial and epicardial wall was analyzed using QuPath v0.6.0-rc3 [https://github.com/qupath/qupath/releases/tag/v0.6.0-rc3].

### Statistics

Variables are presented as mean ± standard error of the mean (SEM). Epicardial activation times within the RVOT, left ventricular free wall, and right ventricular free wall and the number of Purkinje fibers within areas below the pulmonary valve leaflets were compared using a One-Way analysis of variance (ANOVA). Post-Hoc analysis was done with the Bonferroni test. For each statistical test, *p* ≤ 0.05 was considered significant. All statistical analysis were done in SPSS (SPSS Statistics v29.0.0.0 (241); IBM).

## Results

### Delayed activation of the RVOT in the sheep heart

**FIGURE 1:**
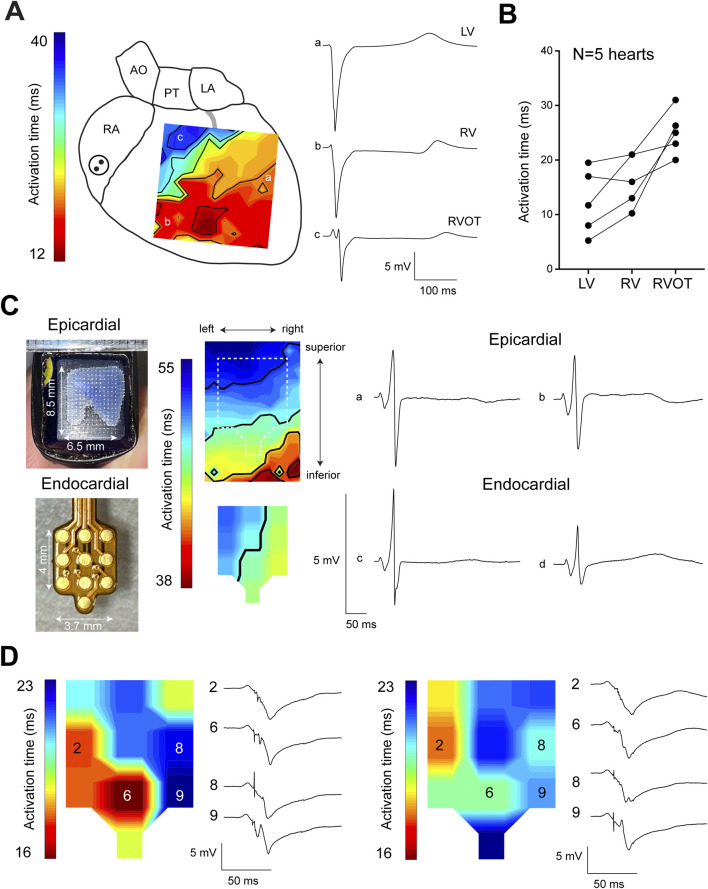
Delayed activation of the sheep RVOT. **(A)** Epicardial activation map of one sheep heart showing late activation of the RVOT. **(B)** Quantitative analysis of the average activation time in the RVOT, right ventricular free wall, and left ventricular free wall. **(C)** Epicardial and endocardial activation maps of the RVOT were simultaneously measured using separate electrode grids. **(D)** Endocardial activation maps of the distal RVOT showing the occurrence of Purkinje spikes preceding myocardial activation. RA, right atrium; AO, aorta; PT, pulmonary trunk; LA, left atrium; RV, right ventricle; LV, left ventricle.

Figure 1A shows an epicardial activation map recorded from the anterior side of a Langendorff-perfused sheep heart, highlighting delayed activation in the RVOT (trace c) compared to the left ventricular free wall (trace a) and right ventricular free wall (trace b). Quantitative analysis of recordings from five sheep hearts revealed that the RVOT was activated, on average, 25.6 ± 1 ms after the onset of ventricular activation. This was significantly later than neighboring parts of the left ventricle and right ventricle which activated at 12.3 ± 2.6 ms and 16.3 ± 2.0 ms, respectively (Figure 1B). To further investigate activation patterns across the RVOT wall, we performed simultaneous epicardial and endocardial mapping of the RVOT just below the pulmonary trunk. Doing so, we found that epicardial activation occurred towards the pulmonary trunk, while the endocardium activated from left to right (Figure 1C). During these measurements we did not detect any Purkinje activity. Next, we continued endocardial mapping by moving the multi-electrode more to inferior into the RVOT. Left and right panels of Figure 1D show two individual activation maps with corresponding electrogram traces. In these electrograms, sharp deflections consistently preceded the larger main deflection, indicative of Purkinje activity. Overall, these findings show functional evidence for Purkinje activity in the sheep RVOT.

**FIGURE 2:**
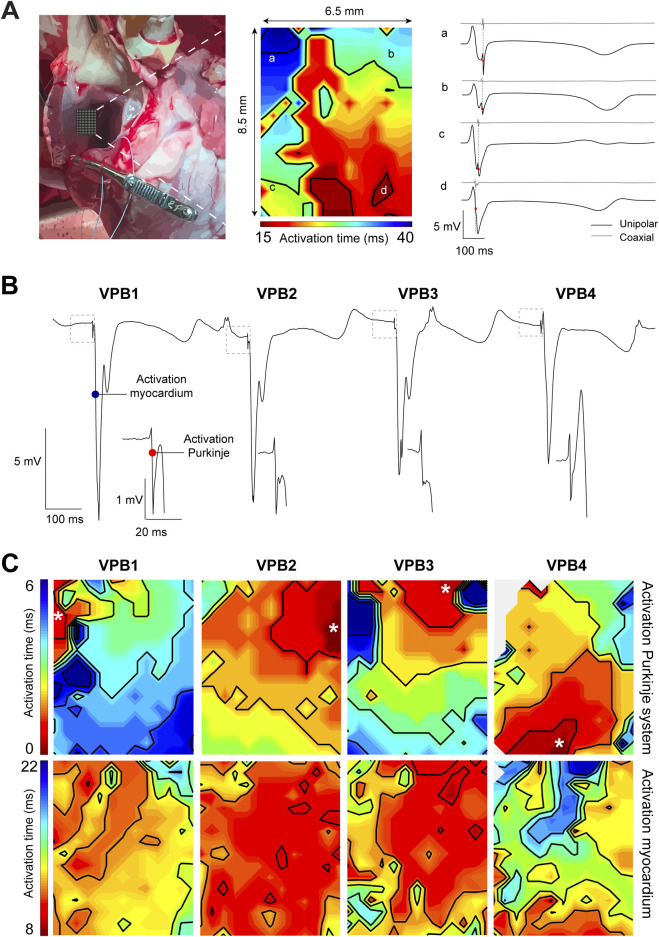
High-resolution endocardial activation mapping of the RVOT reveals Purkinje activity preceding ventricular premature beats (VPBs). **(A)** Left: photograph of the opened right ventricular cavity exposing the endocardial surface of the RVOT free wall. Middle: endocardial activation map of the RVOT recorded during sinus rhythm. Right: corresponding unipolar and coaxial (Hoogendijk et al.) electrogram traces from sites a-d in the activation map. Note the absence of Purkinje spikes in the unipolar electrogram and the single moment of activation in the coaxial electrogram. **(B)** Unipolar electrograms recorded from the RVOT endocardium while moving the catheter multi-electrode. Each VPB was preceded by a Purkinje spike (see insets). **(C)** Local activation maps corresponding to the maximal -dV/dT for Purkinje activation (top row) and myocardial activation (bottom row).

To gain further insight into Purkinje activity from the RVOT endocardium, we opened the right ventricular cavity allowing us to reach luminal side and perform mapping with high-resolution using the 8.5 × 6.5 mm electrode grid previously used for epicardial mapping (see Figure 1). As indicated by an isoelectric ST segment, the opening of the RVOT did not result in ischemia, enabling physiological mapping of the RVOT myocardium (Figure 2A). We identified Purkinje spikes in all five hearts. In one heart, mapping of the exposed RVOT endocardium did not reveal Purkinje activity during sinus rhythm. To confirm that we had only measured the electrical activity of the working myocardium, we calculated coaxial electrograms (Hoogendijk et al.), which revealed a single moment of activation. In this heart, slight movement of the multielectrode catheter along the endocardial surface while applying light pressure initiated VPBs that showed Purkinje activity preceding activation of the working myocardium (Figure 2B). Local activation maps corresponding to the maximal -dV/dt for Purkinje activation (top row) and myocardial activation (bottom row) for each consecutive VPB are shown in Figure 2C. These functional findings show that Purkinje fiber activation from the RVOT endocardium does not necessarily precede activation of the working myocardium. The working myocardium is most likely activated earlier by transmural or subepicardial Purkinje fibers.

### The sheep RVOT contains a Purkinje network that extends into the myocardial wall

**FIGURE 3:**
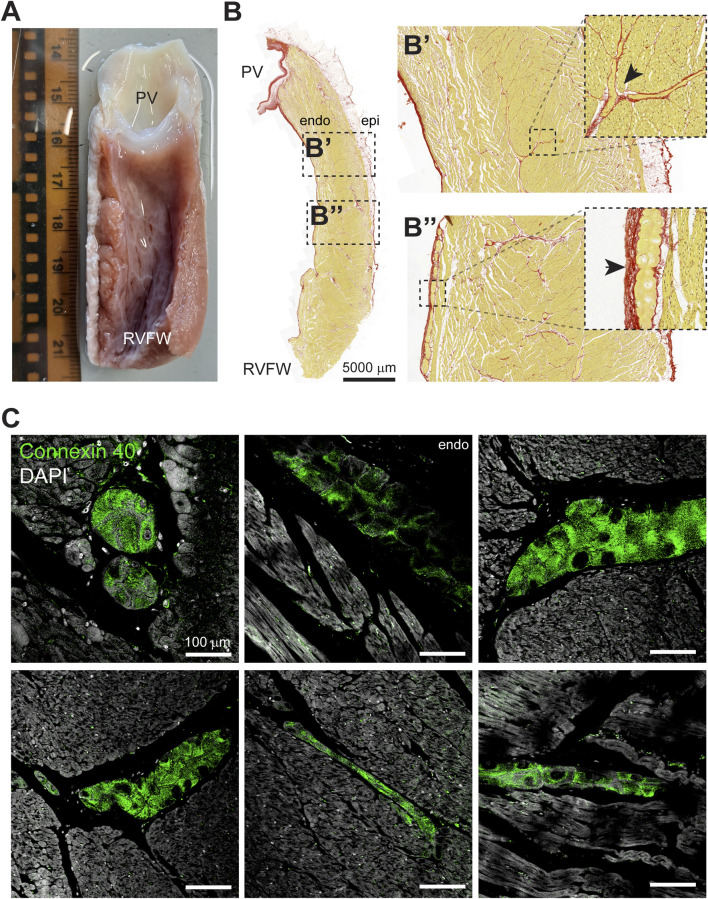
The sheep RVOT contains a Purkinje network that extends into the myocardial wall. **(A)** Photograph of the luminal side of the RVOT free wall after dissection. From this view, the presence of a Purkinje network is clearly visible. Scale in cm. **(B)** Example of a RVOT section stained with Picrosirius Red. Purkinje fibers were identified immediately below the pulmonary valve leaflets **(B’)** and toward the right ventricular free wall **(B”)**. **(C)** Super-resolution microscopy images of Connexin 40-positive Purkinje fibers in the RVOT endocardium and within the myocardial wall.

After the Langendorff experiments, the RVOT free wall was dissected from the heart for the purpose of histological and immunohistochemical examination. On macroscopic level, we observed a dense network composed of grey, flat, gelatinous fibers on the luminal side of the myocardial wall which resembled typical features of the Purkinje system (Figure 3A). The left of Figure 3B displays a dissected RVOT free wall in which histological examination revealed bundles of presumably Purkinje fibers within the subendocardium and deeper within the myocardial wall (Figure 3B’–3B”). Within the bundles, individual myocardial cells were larger and had a paler cytoplasm, which is indicative of a lower myofibril density (Tawara, 1906; De Almeida et al., 2020). Typically, Purkinje fibers were surrounded by a dense fibrous sheath and showed various configurations including cylinders, ovals, and stellate-shaped structures (Figure 3B). To confirm Purkinje cell identity, we next performed immunohistochemical staining for the Purkinje marker Connexin 40 (Miquerol et al., 2004). Purkinje fibers in the RVOT showed marked Connexin 40 expression, which was not present in the working myocardium (Figure 3C). These Purkinje fibers were located within the subendocardium as well as deeper within the myocardial wall.

**FIGURE 4:**
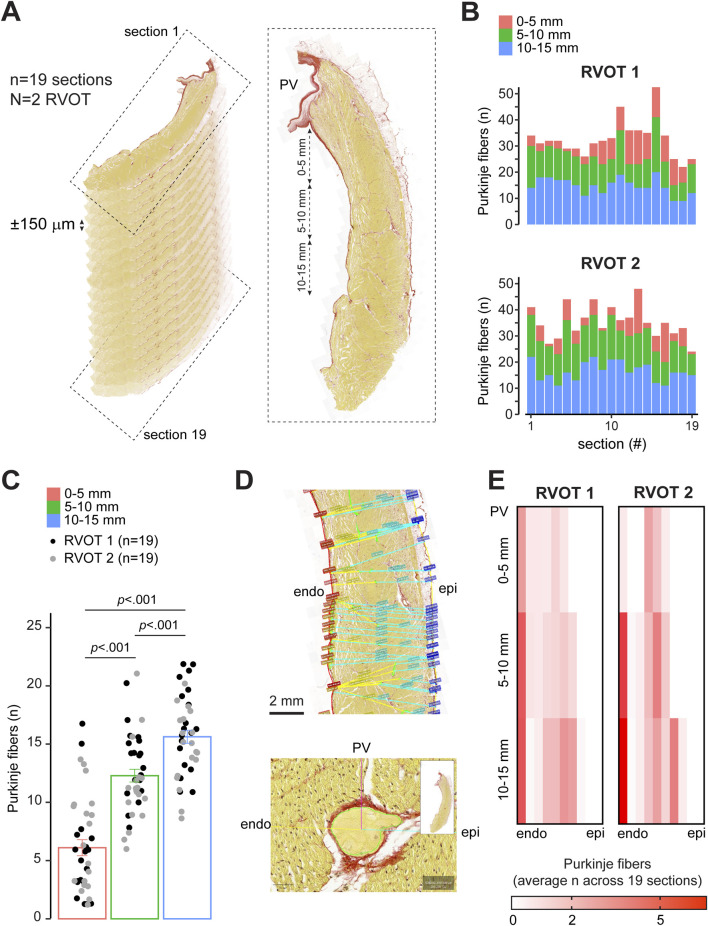
Quantitative analysis of Purkinje fiber distribution in the sheep RVOT. **(A)** Schematic explaining the selection of longitudinal sections from the RVOT free wall. **(B)** Purkinje fiber distribution per section from left-right of the RVOT wall (N = 2) within 0–5 mm, 5–10 mm, and 10–15 mm below the valve. **(C)** Quantitative analysis of Purkinje fiber number within 0–5 mm, 5–10 mm, and 10–15 mm below the valve (n = 38 sections). **(D)** Analysis of the shortest distance from Purkinje fiber to the endocardial (endo) and epicardial (epi) wall. **(E)** Heatmaps of quantitative analysis of the average number of Purkinje fibers for each RVOT between the endocardium and epicardium. Segment width equals 10% RVOT wall thickness.

Although we did measure Purkinje activity on the endocardial side of the RVOT, we did not find it directly below the pulmonary valves. To determine the distribution of Purkinje fibers in the RVOT, we counted the number of individual, insulated Purkinje fibers in longitudinal sections (N = 2 hearts, n = 38 RVOT sections) across two sheep hearts within 0–5 mm, 5–10 mm, and 10–15 mm distance below the valves (Figure 4A). Nineteen sections were chosen from the right to the left side of the RVOT, with an intersection distance of ± 150 μm. We did not observe any major differences in Purkinje fiber count between the left and right side of the RVOT free wall (Figure 4B). However, the number of Purkinje fibers was significantly lower directly below the valves (0–5 mm) than further towards the right ventricular free wall (Figure 4C). Next, we analyzed the shortest distance for each Purkinje fiber relative to endocardium and epicardium for each area (Figure 4D). Figure 4E shows heatmaps for both RVOT free walls in which color intensity corresponds to the average number of Purkinje fibers in each transmural segment (equals to 10% wall thickness) across all 19 sections. Purkinje fiber distribution between endocardium and epicardium was highly similar between both walls. Purkinje fibers were located deeper within the myocardial wall within all areas below the pulmonary valves, but most predominantly when moving closer to more inferior. Altogether, these results confirm the presence of a Purkinje network in the RVOT near the endocardium and extending into the myocardial wall.

## Discussion

We present evidence for the presence of a functional Purkinje system in the RVOT of the sheep heart. Using unipolar mapping, we identified brief sharp spike-shaped deflections preceding the myocardial activation complex. The presence of Purkinje cells was further supported by the a network composed of grey, gelatinous fibers across the RVOT endocardial surface that expressed Purkinje cell marker Connexin 40. This network extended from the right ventricular free wall into the RVOT free wall in close vicinity to the pulmonary valves. The clear presence of a Purkinje network elevates the sheep heart to a model system for studying Purkinje-related RVOT arrhythmia mechanisms.

Our findings are the first to simultaneously provide functional and structural evidence for the presence of a Purkinje network in the RVOT. An earlier study performed by De Almeida et al. demonstrated the presence of a Purkinje system in the RVOT of the cow heart (De Almeida et al., 2020; De Almeida et al., 2017; De Almeida et al., 2021). To do so, the investigators injected Barium-based contrast medium into the Purkinje network and visualized the potential Purkinje system by Computed Tomography. Similarly, we found that the free wall of the RVOT contains a Purkinje fiber network that extends further into the wall. Sheep and cow belong to the ungulates, or hooved mammals, which possess a Purkinje system that extends into the myocardial wall and is more extensive than in human, rodents, and carnivores such as dog (Ansari et al., 1999; Ryu et al., 2009; Ohkawa, 2008; Duan et al., 2017; Elbrønd et al., 2023). A recent study, however, suggests the human ventricular wall may also contain a transmural Purkinje network (Hillestad et al., 2025). The mechanism giving rise to such a transmural Purkinje network is unknown. Research on mice have found that the embryonic ventricular trabecular layer contains precursor cells of the Purkinje network (Christoffels and Moorman, 2009; Jensen et al., 2012). During heart development, some of the cells in the trabecular layer specialize towards a Purkinje phenotype, while others acquire a compact working myocardial phenotype and become part of the compact ventricular wall. In pigs, which are also members of the ungulates, late embryonic hearts have relatively deep intertrabecular recesses and an extensive trabecular layer (Jensen et al., 2024a). This setting appears different from that of human and should the basal parts of this extensive layer undergo compaction further in development, Purkinje precursors could become trapped within the compact wall. Whether such process takes place in pigs and ungulates in general requires further investigation.

The Purkinje fibers we found in the sheep RVOT showed characteristics that aligned with early descriptions (Tawara, 1906; Lhamon, 1912; King, 1916; Hondeghem and Stroobandt, 1974; Canale et al., 1983; Ansari et al., 1999; Ryu et al., 2009). Whether the extension of the Purkinje network into the RVOT is unique to ungulates requires further investigation. Recently, we showed that myocardial trabeculations can exist in the human RVOT. In this study, we found that RVOT trabeculations considerably varied between individuals (Jensen et al., 2024b). Given that Purkinje fibers and trabecular myocardium have a similar origin, this could mean that in some individuals the Purkinje network is present in the RVOT, while in other individuals it is absent.

De Almeida et al. demonstrated the presence of Purkinje fibers in two human RVOT samples using conventional histology along with historic anatomical definitions (De Almeida et al., 2020). To confirm the identity of the Purkinje fibers, we performed an immunohistochemical stain for Connexin 40, a widely accepted marker of the ventricular conduction system in mice that also marks conduction system tissue in the human ventricle (Miquerol et al., 2004; Hillestad et al., 2025). *NPPA* and *MYL4* are expressed during cardiac development and are also specific to the human Purkinje network (Hillestad et al., 2025). The specificity of conventional Purkinje system markers including Contactin 2 (CNTN2) or hyperpolarization-activated channel 4 (HCN4) for the human ventricular conduction system requires further investigation (Pallante et al., 2010).

As reported by De Almeida et al., subendocardial Purkinje fibers in the human RVOT were usually no larger than 50–100 μm in diameter (De Almeida et al., 2020). During endocardial mapping procedures, filter settings in combination with low sampling frequency may hamper detection of electrical activity from these small fibers in patients. An advantage of our experimental Langendorff-perfusion heart model is the possibility to measure electrical activity in the heart without the need to apply filtering settings that are standard in clinical practice. Fundamental improvements in signal processing and filtering technology maybe crucial to identify Purkinje activity for effective catheter ablation of ventricular fibrillation in the RVOT in patients.

In all five sheep we measured, the Purkinje spikes were clearly visible. However, in one heart, Purkinje spikes were only observed following application of light pressure while navigating the electrode catheter against the RVOT endocardial surface. This suggests that the Purkinje system could interfere with baseline RVOT conduction, for example, when the RVOT wall is subjected to increased hemodynamic stresses (Gülan et al., 2019; Hurley et al., 2023a; Hurley et al., 2023b). One such possibility can be examined during studies of the beating heart, in which physiological parameters, including pump function and cardiac pressures, can be measured while blood is pumped through the *ex vivo* heart as would naturally occur (Kappler et al., 2019). Some stretch-activated ion channels, including TRPM4, have been shown to be expressed at higher levels in Purkinje fibers than in ventricular myocardium (Hurley et al., 2023a; Somberg et al., 1981). Activation of these channels produces an inward current that causes diastolic depolarization which enhancing Purkinje automaticity. Mechanical manipulation of Purkinje activity can be avoided by using flexible, stretchable membrane devices for high-resolution electrogram mapping, which are applied directly to the endocardial surface (Gutbrod et al., 2014).

Despite the presence of a Purkinje network, the RVOT does activate later compared to the left and right ventricles. We speculate that this is the result of a longer pathway, originating from the His bundle, that is required for electrical impulse propagation to reach the RVOT free wall compared to the other cardiac regions.

Similar to the RVOT, the Purkinje system is frequently the origin of VPBs (Enriquez et al., 2024). During cardiac repolarization, these VPBs can give rise to polymorphic ventricular tachycardia which may transition into ventricular fibrillation (Haïssaguerre et al., 2002b; Smirk, 1949; Knecht et al., 2009). Currently, it is unknown whether intramural extension of the Purkinje network correlates to a higher susceptibility to idiopathic ventricular fibrillation. However, it is conceivable that a more extensive Purkinje network could give rise to an increased anatomical substrate for reentry (Coronel et al., 2021; Schmitt and Erlanger, 1928).

## Conclusion

The RVOT of the sheep heart contains a functional Purkinje system which can initiate VPBs. This places the sheep heart as an attractive model for experimental research to investigate the role of the Purkinje system in initiation of arrhythmias, including ventricular fibrillation, in the RVOT. Furthermore, the sheep heart could be used as a model for testing novel approaches for Purkinje network ablation.

## Data Availability

The raw data supporting the conclusions of this article will be made available by the authors, without undue reservation.
